# Vitamin C and Phenolic Antioxidants of Jua (*Ziziphus joazeiro* M.) Pulp: A Rich Underexplored Brazilian Source of Ellagic Acid Recovered by Aqueous Ultrasound-Assisted Extraction

**DOI:** 10.3390/molecules27030627

**Published:** 2022-01-19

**Authors:** Thaís Silva da Rocha, Alessandro de Lima, Jurandy do Nascimento Silva, Geni Rodrigues Sampaio, Rosana Aparecida Manólio Soares Freitas, Renan Danielski, Adriano Costa de Camargo, Fereidoon Shahidi, Elizabeth Aparecida Ferraz da Silva Torres

**Affiliations:** 1 Department of Nutrition, Universidade Federal do Piauí-UFPI, Teresina 64049-550, PI, Brazil; thais.rocha@ifma.edu.br; 2 Instituto Federal de Educação, Ciência e Tecnologia do Piauí-IFPI-Zona Sul, Teresina 64000-040, PI, Brazil; alessandro@ifpi.edu.br (A.d.L.); jurandy@ifpi.edu.br (J.d.N.S.); 3 School of Public Health, Universidade de São Paulo—USP, São Paulo 01246-904, SP, Brazil; genirs@usp.br (G.R.S.); rosanaso@usp.br (R.A.M.S.F.); 4 Department of Biochemistry, Memorial University of Newfoundland, St. John’s, NL A1C 5ST, Canada; rdanielski@mun.ca (R.D.); fshahidi@mun.ca (F.S.); 5 Nutrition and Food Technology Institute, University of Chile, Santiago 7830490, Chile

**Keywords:** polyphenols, ellagic acid, gallic acid, ultrasound-assisted extraction, underexplored fruits

## Abstract

Jua (*juá* in Portuguese) is an underexplored fruit from Brazil’s northeast. This fruit is rich in antioxidant substances. However, there is a dearth of information about jua’s bioactive potential. The present study evaluated two extraction methods (continuous agitation and ultrasound-assisted extraction—UAE) and employed three different solvents (water, ethanol, and acetone) to efficiently recover soluble phenolic compounds. Aqueous extracts obtained by UAE showed the highest total phenolic content (TPC) and antiradical activity. Besides being an eco-friendly procedure, extraction and/or solubility in an aqueous medium is also important for food application. Ellagic acids were the predominant phenolics (80%) found in aqueous jua pulp extract obtained by UAE, as determined by HPLC, while its TPC was 405.8 gallic acid equivalent per gram of fruit. This extract also exhibited a higher scavenging activity towards peroxyl radicals when compared to that of several other fruits from the literature, including grape, strawberry, cranberry, and walnuts, which are known references in terms of antioxidants. This is the first report that demonstrates jua pulp’s potential as an alternative source of ellagic acid and other phenolic acids and flavonoids. Therefore, the outcome of this study provides new information that can be useful for functional food and nutraceutical industries.

## 1. Introduction

**Figure 1:**
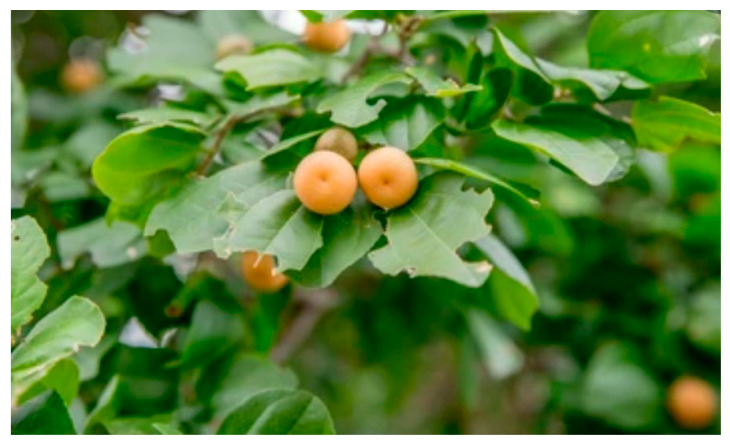
Juá (*Ziziphus joazeiro* M.) fruit. Source: https://www.shutterstock.com/pt/image-photo/fruits-de-boi-1076258687 (accessed on 15 January 2022).

The juazeiro tree (*Ziziphus joazeiro* M.), which belongs to the family *Rhamnaceae,* grows in the Brazilian northeast and is characterized for thriving under adverse climatic conditions encountered in the *caatinga* desert, namely hot and dry winter as well as cold and rainy summer. Parts of the tree, such as the leaves, are used in folk medicine to treat respiratory conditions, such as asthma, pneumonia, and bronchitis, as well as other ailments (e.g., headache, fever, skin rash) [1]. The plant bears small round-shaped fruits, known as jua, possessing a yellow color, sweet taste, and a seed in its core (Figure 1). Although used by the local population, scientific data on jua’s chemical composition is scarce. Evidence points out that the fruit is highly concentrated in bioactive compounds, including vitamin C and phenolic compounds [1,2].

Health-promoting benefits have been ascribed to the consumption of fruits and vegetables [3], which, among other factors, could be associated with the presence of phenolic compounds. This large group of plant secondary metabolites is characterized for their ability to scavenge free radicals primarily by the donation of a hydrogen atom or an electron, interrupting the propagation of oxidation [4]. Oxidative stress can occur in the body when there is an accumulation of reactive oxygen species (ROS), and this condition is related to the development of several chronic ailments, such as cardiovascular diseases, some types of cancer, diabetes, obesity, and anti-inflammatory disorders. The consumption of antioxidants, such as phenolic compounds, along with a healthy lifestyle, may help in lowering the risk of developing these diseases [5,6].

In order to fully understand the potential of underexplored antioxidant sources such as jua, it is necessary to extract the relevant bioactive compounds from the fruit and analyze their composition. For instance, soluble phenolics can be obtained by several techniques, each having its own advantages and pitfalls. Among possible approaches, ultrasound-assisted extraction (UAE) stands out for its simplicity, economical apparatus, low solvent use, and the possibility of simultaneously conducting several experiments. Besides, the time required for each extraction is relatively short, ranging from 20 and 40 min on average [7]. 

UAE relies on the acoustic cavitation phenomenon, by which the formation and collapse of microbubbles generate specific sites with extreme temperature (5000 K) and pressure (1000 atm), causing higher shear and turbulence in the cavitation area. This effect helps disrupt the plant tissue, which increases the rate of solvent penetration. Additionally, the ultrasonic waves promote hydration and swelling of the material, increasing the pore size and creating a sponge-like effect, which optimizes the interchange between liquids from inside the pores and the matrix’s surface, enhancing mass transfer [8,9]. According to Ma et al. [10], UAE has also attracted commercial interest over the years due to its reduced extraction time, cost-effectiveness, the possibility of scaling-up, and reduced solvent use.

Especially for new or underexplored feedstocks, an appropriate solvent should be chosen to ensure the dissolution and full extraction of the target compounds. For phenolic compounds, water, methanol, ethanol, and acetone are the most common solvents used either alone or in combination [8]. Therefore, the objective of this study was to explore the chemical composition of jua, with an emphasis on its phenolic composition and antiradical activity towards peroxyl radicals. In addition, for the first time, combinations of two extraction methods (continuous agitation and ultrasound-assisted extraction) with three solvents (water, ethanol, and acetone) were employed to investigate the best conditions for the highest extraction efficiency of soluble phenolic compounds. 

## 2. Results and Discussion

### 2.1. Vitamin C

Ascorbic acid (vitamin C), a vitamin antioxidant, was present at 87.28 mg/100 g in jua pulp. This value is higher than those of common fruits such as apple (5.7 mg/100 g), red grape (4.0 mg/100 g), strawberry (37 mg/100 g), lemon (46 mg/100 g), peach (6.6 mg/100 g), orange (45 mg/100 g), banana (9.1 mg/100 g), pear (4.0 mg/100 g), pineapple (15.4 mg/100 g), and grapefruit (37 mg/100 g) [11]. As humans cannot synthesize this compound, it needs to be acquired through the diet. In light of this, jua pulp has been shown to be a rich source of vitamin C, contributing to the fruit’s health benefits.

Ascorbic acid is a water-soluble vitamin of utmost biological significance. Besides preventing the common cold, some other roles assumed by ascorbic acid include participation in the metabolism of tyrosine, folic acid, and tryptophan, lowering of cholesterol, contribution to the synthesis of carnitine and catecholamine to regulate the nervous system, tissue growth and wound healing, among others. Besides, as an antioxidant molecule, this vitamin can retard oxidative stress, and it has been related to anticancer and anti-diabetic effects [12]. 

Due to the antioxidant nature of ascorbic acid, in vitro assays used to estimate the antioxidant activity are also affected by this molecule’s presence in case of it being co-extracted with other antioxidants, such as phenolics [12]. Therefore, it is important to quantify the amount of vitamin C, so the results can faithfully reflect the compounds that contribute to the samples’ antioxidant potential.

### 2.2. Effect of Extraction Method on Total Soluble Phenolic Content

In order to evaluate how the solvent system and the extraction method can affect the phenolic content of jua pulp, the extraction was conducted through continuous agitation (CA) and ultrasound-assisted extraction (UAE), using water, ethanol (logP of −0.31), or acetone (logP of −0.24) as solvents. Results for the extracts’ total phenolic contents (TPC) are shown in Table 1. 

The combination of UAE with water (405.8 mg GAE/100 g) and/or ethanol (394.9 mg GAE/100 g) rendered the highest total phenolic content from all the combinations tested, followed by the extract obtained using acetone and UAE (275.1 mg GAE/100 g). Therefore, UAE proved to be a more efficient extraction technique when compared with continuous agitation, obtaining higher phenolic yields. 

The use of ultrasound as an extraction technique can yield compounds not possible to be released by conventional approaches, such as maceration. Lima [13] was able to recover a total of 37 phenolic compounds from guava’s pulp and processing by-products by using UAE, including ellagic acid, gallic acid, quercetin, as well as phenolics never reported before for this fruit, such as umbelliferone, carnosol, and syringaldehyde. Mazza [14] also reported a positive outcome when using ultrasound extraction to obtain phenolics from syrah grape skin. Their optimization study revealed that an ultrasonic power of 3000 W/L, 2.5% citric acid, and solid:liquid ratio of 1:15 yielded the highest levels of total phenolics, monomeric anthocyanins, and antioxidant capacity measured by ORAC and ABTS assays. 

### 2.3. Identification of Phenolic Compounds by High-Performance Liquid Chromatography (HPLC)

Table 2 shows the phenolic compounds identified in jua pulp extracts by HPLC analysis, with their respective quantities. Overall, the main compounds present in the aqueous extract obtained by UAE (highest total phenolic content) were ellagic acid, followed by gallic acid and epicatechin. Ellagic acid is a potent antioxidant, mainly due to the presence of multiple hydroxyl groups in the ortho position in the molecule, contributing to its ability to donate hydrogen atoms, and stabilizing free radicals. Further studies may be focused on optimizing the conditions (e.g., temperature, extraction time, pH, etc.) to increase the recovery of ellagic acid as the most prominent compound [15]. 

Jua pulp was found to be richer in soluble free ellagic acid (974 µg ellagic acid/g DW). Although Kakadu plum exceeds this level, the concentration of ellagic acid in jua remains higher than other commercialized fruits, such as strawberry (630 µg ellagic acid/g DW), cranberry (120.0 µg ellagic acid/g DW) and pomegranate [mesocarp, 234.2 ± 13.0 (dw) and peel 637.7 ± 32.8 (dw)]. Walnuts (590 ellagic acid/g DW) are also popular sources of ellagic acid [16,17,18]. As a phenolic compound, ellagic acid can dampen oxidative stress, promoting numerous health benefits. For instance, this substance has been related to the prevention of cancer, diabetes, obesity, cardiovascular, gastrointestinal, and neurodegenerative diseases, among others. Therefore, plant material and agro-industrial by-products can serve as sources for the isolation of ellagic acid, which could be further purified and used in several areas, including cosmetics, feed formulation, nutraceuticals, and the food industry, acting as a natural antioxidant. The isolation and purification of this molecule can be performed by using conventional techniques, such as acid-solvent extraction with concentrated hydrochloric acid and methanol, or by more eco-friendly approaches. UAE, used in the present study, is an example of the latter, due to its low solvent usage, economical effectiveness, and reduced extraction time [19].

Since the extraction procedure does not render high selectivity, the recovery of a target compound (e.g., ellagic acid) needs to be followed by a purification step. Adsorption/desorption is widely used for purifying phenolic compounds. It involves the addition of the crude extract containing the target molecule through a column filled with the adsorbent material. Then, a desorption step is conducted with organic solvents (e.g., methanol, ethanol) to recover the phenolic of interest. Several adsorbents can be used, including activated carbon, resins, and polysaccharides, among others. Other techniques can be coupled with adsorption/desorption in order to further increase the compound’s purity, such as ultrafiltration and nanofiltration, which work by size exclusion [20,21].

Therefore, the high concentration of ellagic acid in jua pulp extracts may render a significant contribution to its bioactive properties, classifying jua as an alternate source for the recovery of ellagic acid. Other sources of ellagic acid include pecans [22], Brazil nuts [23], fennel seed [24], mango seed, longan seed, pomegranate peel, walnuts’ skin, and other underutilized fruits such as Canarium odontophyllum, Dacryodes rostrata, Mangifera pajang, and Parkia speciosa [25,26,27,28]. 

Gallic acid was the second most abundant compound, regardless of the solvent employed. The structural features of gallic acid, with three hydroxyl groups, also contribute to the strong antiradical activity of the molecule. Additionally, gallic acid has the ability to form inter- and intra-molecular hydrogen bonds, contributing to the stability of the compound. Similar to ellagic acid, the number of hydroxyl groups, free of steric hindrance, renders a high radical scavenging activity. The ability of gallic acid to minimize the excessive production of reactive oxygen species may result in a lower risk of developing neurodegenerative conditions, such as Parkinson’s and Alzheimer’s disease [29].

The HPLC-TPC (sum of all phenolics quantified in each extract obtained upon UAE) demonstrated that the aqueous and ethanolic extracts are richer in polyphenol content than the acetone extract. This result is in accordance with the trend previously observed for TPC values obtained by the spectrophotometric analysis (Table 1), lending support to the fact that water and ethanol are more efficient than acetone in extracting phenolic compounds from jua pulp under the experimental conditions employed. However, besides being an eco-friendly procedure, an aqueous medium is of great importance for food application. 

### 2.4. Antiradical Activity

Table 3 shows the antiradical activity results of jua pulp extracts.

Similar to the trend observed for TPC, aqueous extracts obtained by UAE exhibited a higher scavenging activity towards peroxyl radicals (236.0 ± 10.80 µmol TE/100 g fresh weight) than the extract obtained by CA (161.5 ± 5.10 µmol TE/100 g fresh weight). The ORAC values of jua pulp obtained by UAE on a dry weight basis (10,926 µmol TE/100 g) were similar or higher than those of several fruits from the literature (e.g., fig, kiwi, lucuma, mango, melon, avocado, pineapple, banana, watermelon), including some types of grapes [30], which are references when it comes to the presence of phenolic antioxidants. Grapes, their products, and processing by-products have attracted much attention in the field. A recent study demonstrated that the ability of phenolic extracts in scavenging peroxyl radicals (ORAC method) led to being able to anticipate the biological properties of the test material in cell models [31]. Therefore, it is reasonable to suggest that, by showing higher ORAC values, jua pulp may provide greater health benefits than the mentioned fruits, although this assumption remains to be confirmed in animal models and/or human studies. Considering the importance of aqueous extraction in the context of green chemistry and food safety, the UAE was repeated using water, ethanol, and acetone and evaluated by a different method (ABTS radical cation) for confirmation. The results are shown in Table 4. 

Lending support to the finding obtained for TPC, UAE rendered the highest TEAC values (1500 ± 109 µmol TE/100 g fresh weight), while no significant difference was found between ethanol (840 ± 59 µmol TE/100 g fresh weight) and acetone (790 ± 79 µmol TE/100 g fresh weight). These results are in agreement with some of the TEAC results reported by Rufino [33] when studying several exotic fruits from Brazil. Açaí (1510 µmol TE/100 g), cajú (1120 µmol TE/100 g), carnaúba (1070 µmol TE/100 g), and mangaba (1460 µmol TE/100 g) showed similar findings to those reported in the present studies. Meanwhile, other fruits such as cajá (780 µmol TE/100 g) and umbu (630 µmol TE/100 g) were found to possess lower TEAC values than those of jua pulp extracts.

As mentioned earlier, ellagic acid was the main phenolic present in jua pulp (Table 2). The ability of different sources of ellagic acid to mitigate oxidative stress has also been reported owing to its scavenging activity towards hydroxyl radicals. This has also been related to their evidenced in vitro protection against DNA-damage and LDL-cholesterol oxidation, which are accepted as biomarkers for potential cancer development and risk of cardiovascular diseases, respectively [26,27]. Likewise, the anti-inflammatory effects of ellagic acid are supported by literature data. According to Chen et al. [34], ellagic acid effectively decreased IL-6 protein expression and cytokine release through inactivation of JNK and p65 pathways. Finally, the abilities of some sources of ellagic acid in inhibiting the activity related to the absorption of carbohydrates and lipids (e.g., alpha-glucosidase) and potential prevention of type 2 diabetes and obesity, as well as acetylcholinesterase, which has a key role in the development of Alzheimer’s disease, have been reported [26,27,35].

## 3. Materials and Methods

### 3.1. Samples and Chemicals

Jua fruits (*Ziziphus joazeiro* M.) were collected from the city of Santa Rosa (Piaui, Brazil). The phenolic standards, namely phenolic acids (chlorogenic, caffeic, gallic, vanillic, trans-cinnamic, protocatechuic, hydroxybenzoic, ferulic, benzoic, salicylic, o-coumaric, p-coumaric, sinapic, gentisic, quinic, and ellagic acids), flavan-3-ols (catechin, epicatechin), flavonol (quercetin), and flavone (apigenin), were purchased from Sigma-Aldrich (São Paulo, Brazil). All other chemicals used in this study were obtained either from Sigma-Aldrich (São Paulo, Brazil), Carlo Erba (CER, São Paulo, Brazil), or Merck (Darmstad, Germany).

### 3.2. Determination of Vitamin C

Ascorbic acid (vitamin C) was analyzed by the Tillmans method, based on the reduction of 2,6-dichlorophenol indophenol sodium salt (DISS) by ascorbic acid [36]. First, an ascorbic acid standard solution (10 mL) was mixed with an oxalic acid solution (50 mL). Then, the resulting mixture was titrated with the DISS solution. Subsequently, the same procedure was repeated by replacing the ascorbic acid solution with the samples. The amount of ascorbic acid was calculated according to the following equation:

$$\mathrm{Ascorbic}\;\mathrm{acid}\;\left(\mathrm{mg}/100\;\mathrm{mL}\right)=\frac{V\times F\times 100}{A}$$

where *V* is the volume of Tillmans solution used in the titration, *F* is the correction factor of the Tillmans solution, and *A* is the sample volume (mL).

In order to calculate the correction factor (*F*), the amount of vitamin C (mg) used in the titration was divided by the volume of Tillmans solution used (mL).

### 3.3. Phenolic Extraction

Fresh fruit samples were used to obtain aqueous (distilled water), ethanolic (100% ethanol), and acetone extracts (100%) [37]. The samples (5 g) were mixed with 50 mL of solvent and homogenized with an Ultra-turrax dispenser for 1 min. The mixtures were then submitted to either continuous agitation (CA) at 25 °C for 1 h or ultrasound-assisted extraction (UAE—ELMA, Elmasonic P 60 H, Singen, Germany), at a frequency of 37 kHz, temperature of 25 °C, for 1 h. Subsequently, the extracts were centrifuged at 3000× *g* rpm for 10 min at 20 °C. The supernatant was collected and stored in Ambar flasks under refrigeration (±4 °C) until further analysis.

### 3.4. Total Phenolic Content of Soluble Extracts

This determination was performed according to Swain and Hillis [38]. Sample extracts (0.5 mL) were mixed with 8 mL of distilled water and 0.5 mL of Folin–Ciocalteu reagent. The content was homogenized, and after 3 min, 1 mL of a saturated sodium carbonate solution was added to the reaction tube. The mixture was allowed to react for 1 h in the dark, and the absorbance was read at 720 nm. Total phenolic content was expressed as mg of gallic acid equivalents (GAE)/100 g of sample.

### 3.5. Identification of Phenolic Compounds by High-Performance Liquid Chromatography (HPLC)

The HPLC analysis of jua extracts was performed according to previous studies [39,40]. The HPLC apparatus (Shimadzu, LC20AT, Kyoto, Japan) consisted of an automated sample injector (SIL-20AC), controller (CBM-20), column oven (CTO-20), and diode array detector (SPD-M20A). A C18 stationary phase column (Shimadzu, Shim-Pack, VP-ODS-2, 25 × 0.6 cm, particle of 5 µm) was used. The mobile phase was composed of 0.1% (*v*/*v*) deionized water (A) and acetonitrile (B) in a proportion of 9.5 to 0.5 of A to B. The gradient elution followed the order: 5% B (10 min), 5–100% B (40 min), 100% B (5 min). The oven temperature was kept at 35 °C, and the elution rate was set at 1 mL/min during the analysis. The detection was performed between 190 and 400 nm. The coefficients of determination (R2) ranged from 0.9925 to 0.9999. The limits of detection (LOD) were in the range of 0.2 to 164.1 ng·mL^−1^, while the limits of quantification (LOQ) ranged from 0.7 to 547.1 ng·mL^−1^. 

### 3.6. Antiradical Activity towards Peroxyl Radicals

For this determination, the protocol reported by Ou et al. [41], as adapted by Prior et al. [42], was followed. Prior to analysis, the fluorescein reagent was diluted with a phosphate buffer solution. An APPH [2,2′-azobis(2-methylpropionamidine) dihydrochloride] solution was also prepared with phosphate buffer. Subsequently, 50 µL of sample was added to a microplate along with 150 µL of fluorescein solution. The microplate was shaken and allowed to stand for 15 min at 37 °C, with a further addition of 50 µL of AAPH solution. The microplate was once again shaken and allowed to react for 60 min. The fluorescence detection (excitation: 485 nm/emission: 535 nm) was then read in a microplate reader (Molecular Devices, Sunnyvale, CA, USA). The results were expressed as µmol Trolox equivalents per 100 g of fruit (µmol TE/100 g of fruit).

### 3.7. Trolox Equivalent Antioxidant Capacity (TEAC)

An ABTS^•+^ solution was prepared by combining 7 mM of ABTS with 2.45 mM of potassium persulfate. The solution was allowed to react in the dark for 12 h, being further diluted with ethanol until an absorbance value of 0.70 ± 0.01. The extract samples were also diluted with ethanol (1:5, *v*/*v* and 1:10, *v*/*v*). Subsequently, 40 µL of each diluted sample was mixed with 1960 µL of a freshly prepared radical solution. The mixture reacted for 6 min, and the absorbance was read at 734 nm. Trolox solutions in ethanol were used as standards at concentrations of 20 and 50 µM. Ethanol alone was used as a control [43].

### 3.8. Statistical Analysis

The data (n = 3) were analyzed by using the software ASSISTAT (7.6 beta). The analysis of variance (ANOVA) was calculated, and the means were compared using the Tukey test (*p* < 0.05).

## 4. Conclusions

Aqueous extracts obtained by ultrasound-assisted extraction showed the highest efficiency in the recovery of soluble phenolics, as supported by their total phenolic content, which may be attributed to the cavitation phenomenon promoted by the ultrasound. The HPLC analysis of jua pulp extracts showed that it was mainly composed of phenolic acids, ranging from 91% to 94%, depending on the extraction solvent (water, ethanol, and acetone). Ellagic acid, which is known for its potent radical scavenging activity, was the most abundant phenolic (80% in the aqueous extract). The higher antiradical activity of the aqueous extracts obtained by UAE was confirmed by its ability to scavenge peroxyl radicals. This is the first report that demonstrates that jua can be a better source of ellagic acid than traditional sources such as strawberry, cranberry, and walnuts. Future studies should concentrate on the effects of food processing, digestibility, bioaccessibility, and bioavailability, and further biological activities of the fruit and its industrial products in animal models and/or clinical trials. For this, ellagic acid must be used as a target compound. Especially in the nutraceutical industry, which frequently deals with concentrated compounds and/or mixtures, toxicity testing must be contemplated. 

## Figures and Tables

**Table 1:** Total soluble phenolic content (mg GAE/100 g FW) of jua (*Ziziphus joazeiro* M.) obtained by continuous agitation (CA) and ultrasound-assisted extraction (UAE) using different solvents.

Solvent	CA	UAE
Water	338.7 ± 29.00 Ab	405.8 ± 6.01 Aa
Ethanol	314.2 ± 12.45 Ab	394.9 ± 24.4 Aa
Acetone	271.5 ± 1.78 Ba	275.1 ± 15.87 Ba

GAE, gallic acid equivalent; FW, fresh weight. Results are expressed as mean (n = 3) ± standard deviation (SD). Results followed by the same lowercase letter in the row and the same uppercase letter in the column do not differ significantly (Tukey’s test, *p* < 0.05).

**Table 2:** Phenolic compounds in jua pulp extracts obtained upon ultrasound-assisted extraction.

Phenolic Compound	Extracts (µg/g FW) *		
	Water	Ethanol	Acetone
Phenolic acids			
Gallic acid	29.30 ± 2.8 aA	32.66 ± 2.6 aA	16.50 ± 1.4 bA
*p*-Coumaric acid	0.64 ± 0.1 aB	0.58 ± 0.1 bB	0.99 ± 0.10 aB
Sinapic acid	5.56 ± 0.2 aB	3.48 ± 0.3 bB	0.82 ± 0.2 cB
Ellagic acid	210.44 ± 20.1 aC	210.97 ± 17.1 aC	192.13 ± 14.3 aC
Flavan-3-ols			
Catechin	4.51 ± 1.1 aB	6.14 ± 0.3 aB	12.55 ± 1.7 bA
Epicatechin	9.23 ± 0.4 aAB	7.56 ± 1.2 abB	6.30 ± 0.80 bAB
Flavonol			
Quercetin	1.62 ± 0.1 aB	1.54 ± 0.2 aB	1.27 ± 0.3 aAB
Total (µg/g FW)	261.30	262.93	230.56

* Results are expressed as mean (n = 3) ± standard deviation (SD). Results followed by the same lowercase letter in the row and the same uppercase letter in the column do not differ significantly (Tukey’s test, *p* < 0.05).

**Table 3:** Antiradical activity results for jua pulp extracts obtained by ultrasound-assisted extraction and continuous agitation, and oxygen radical absorbance capacity of selected fruits obtained from the literature.

	Jua		Kiwi	Fig	Lucuma	Mango	Melon	Avocado	Pineapple	Banana	Watermelon	Grape
	Ultrasound-Assisted Extraction *	Continuous Agitation *										
Peroxyl radical scavenging activity, ORAC assay (µmol TE/100 g)	236 ± 18.80 ^a^ f.w.	161.5 ± 5.10 ^b^ f.w.	-	-	-	-	-	-	-	-	-	-
Oxygen radical absorbance capacity (µmol TE/100 g) [30]	10,926 ± 870 d.w.	7475 ± 236 d.w.	952–5860 d.w.	953–6332 d.w.	533–1152 d.w.	322–1822 d.w.	128–2299 d.w.	912–19,127 d.w.	968–7223 d.w.	1528–20,922 d.w.	193–1708 d.w.	1085–19,819 d.w.

* Results are expressed as mean (n = 3) ± standard deviation (SD). Results followed by the same lowercase letter in the row and the same uppercase letter in the column do not differ significantly (Tukey’s test, *p* < 0.05).

**Table 4:** Trolox equivalent antioxidant capacity (TEAC, µmol TE/100 g fw) of jua pulp extracts obtained by ultrasound-assisted extraction with water, ethanol, and acetone compared with results for methanol extracts from exotic fruits obtained from the literature.

	Water	Ethanol	Acetone	Methanol [32]
Jua	1500 ± 109	840 ± 59	790 ± 79	
Ciruela	-	-	-	625 ± 4
Jackfruit	-	-	-	63 ± 1
Mangaba	-	-	-	1084 ± 13
Murici	-	-	-	1573 ± 1
Papaya	-	-	-	760 ± 20
Pineapple	-	-	-	378 ± 3
Sapodilla	-	-	-	99 ± 11
Soursop	-	-	-	609 ± 13
Sweetsop	-	-	-	621 ± 62
Tamarind	-	-	-	832 ± 11
Umbu	-	-	-	107 ± 0

Results are expressed as mean (n = 3) ± standard deviation (SD). Results followed by the same lowercase letter in the row and the same uppercase letter in the column do not differ significantly (Tukey’s test, *p* < 0.05).

## Data Availability

The data presented in this study are available on request from the corresponding author.

## Sample Availability

Samples of the compounds are not available from the authors.
